# Effect of Low-Dose Atropine on Choroidal Thickness in Children With Myopia Progression

**DOI:** 10.7759/cureus.88047

**Published:** 2025-07-16

**Authors:** Shrutakirty Parida, Matuli Das, Snehalata Dash, Shoubhik Chakraborty, Soumyakanta Mohanty, Shovna Dash

**Affiliations:** 1 Pediatric Ophthalmology, Strabismus and Neuro-ophthalmology, Kalinga Institute of Medical Sciences, Bhubaneswar, IND; 2 Ophthalmology, Kalinga Institute of Medical Sciences, Bhubaneswar, IND; 3 Ophthalmology, SUM Hospital, Bhubaneswar, IND

**Keywords:** axial length, best corrected visual acuity, choroidal thickness, enhanced-depth imaging, intraocular pressure, oct (optical coherence tomography), retinal pigment epithelium (rpe), spherical equivalent (se)

## Abstract

Purpose: The present study aims to evaluate the effect of low-dose atropine (0.01%) on choroidal thickness in children with progressive myopia. A secondary objective was to compare the rate of myopia progression between children treated with low-dose atropine (0.01%) and those receiving placebo eye drops (preservative-free carboxymethyl cellulose 0.5%) through changes in equivalent, axial length (AXL), and best-corrected visual acuity (BCVA). Study design: A prospective case-control interventional study was conducted in the department of ophthalmology at a tertiary eye care center in eastern India.

Materials and methods: A total of 87 children aged 5-16 years with bilateral progressive myopia were recruited and randomly assigned into two groups. Spherical equivalence, AXL, and choroidal thickness (sub-foveal and at 1500 and 3000 microns nasal and temporal to the fovea) were documented at baseline,1 month,3 months, and 6 months.44 children in group A received treatment with once-daily dosing of atropine at bedtime, while 43 children in group B received a placebo eyedrop.

Results: Children in group A showed a significant increase in overall choroidal thickness at 3 and 6 months (11+/-9.67) and (18+/-13.43) microns, respectively, which showed a significant correlation with the progression of myopia (in terms of spherical equivalence and AXL).

Conclusion: Low-dose atropine induced a significant choroidal thickening effect, which was associated with slower progression of myopia in the treatment group.

## Introduction

The rapid rise in the prevalence of myopia has become a major public health problem globally [1]. It has been predicted that nearly half of the world population will develop myopia by the year 2050 [2,3]. It is extremely important to retard the development of myopia due to the high risks associated with pathological myopia and its ocular complications. Optical and surgical procedures for correcting refractive errors fail to arrest the development of progressive myopia, even if they correct it. Techniques to retard the progression of myopia include application of progressive addition bifocal glasses/lenses, peripheral defocus altering lenses, other types of contact lenses (such as multifocal and orthokeratology), additional time spent outdoors, and some drugs with pharmacological activity [4,5,6]. Atropine eye ointment has for a while now been investigated for its promising role in myopia management, with pioneering efforts being contributed by Bedrossian and Kelly [7,8], Gimbel and Yen [9,10]. Previous literature suggests that atropine reduces the prevalence of myopia progression as well as axial elongation of eyes by non-accommodatory methods.

Gong and collaborators have demonstrated that atropine is equally efficacious in the concentration range of 0.01% to 1% while with increasing concentrations, adverse effects might get magnified [11]. The drug has also been found to thicken the choroid in children through presumable dopamine-mediated mechanisms involving muscarinic acetylcholine receptors that have been related to lateral compression of axial elongation and slower development of myopia [12]. Moreover, modulation of retinal and scleral muscarinic receptors can have an effect on the extracellular matrix of the sclera. These receptors regulate scleral fibroblast activity and are postulated to be involved in structural remodeling that underlies progressive myopia [12,13]. In addition to receptor-mediated effects, atropine can exert a direct effect upon scleral fibroblasts and has been reported to inhibit glycosaminoglycan synthesis with resultant prevention of ocular elongation [13,14]. It has also been postulated that low-dose atropine exerts its effect through M1 and M4 receptors in the retina [14]. The overall therapeutic effect of atropine can therefore be postulated to be of a multifactorial nature, with several of these biological processes included.

Choroid, being the vascular layer of the eye, plays a significant role in supplying nutrients to the retina, maintaining intraocular pressure (IOP), establishing eye temperature, and regulating ocular development [15,16]. Retinal defocus has been reported to have an impact on choroidal thickness (ChT) and has been implicated in myopia development and progression [16,17]. Choroidal thickness has also been reported to be disrupted in several retinal and choroidal diseases, including age-related macular degeneration, central serous chorioretinopathy, diabetic retinopathy, and glaucoma [16,17]. Enhanced Depth Imaging Optical Coherence Tomography (EDI- OCT) has improved choroid-scleral interface visualization and has facilitated measurement of ChT with high precision. Sub-foveal choroidal thickness has been defined as being from the outer border of hyperreflective retinal pigment epithelium to the inner border of hyperreflective suprachoroidal space. It is taken perpendicular to a tangential line to the foveal surface. Sub-foveal measurement of ChT has been reported to be correlated with age, gender, axial elongation of eyes, IOP, and systemic blood pressure [17,18].

## Materials and methods

This was a prospective single-blind interventional case-control study conducted at the department of ophthalmology in a tertiary eye care hospital, where the participants were unaware of the group allocation. The study was conducted according to the principles of the Declaration of Helsinki and was approved by the ethical committee. All the participants and their parents gave their informed consent before the study. Computer-generated randomization was done. One hundred ten participants (55 cases and 55 controls) aged 5-16 years with bilateral progressive myopia were recruited, out of which 44 cases and 43 controls were included, and the remaining participants were excluded as per the exclusion criteria. We excluded patients who were allergic or hypersensitive to atropine, had a poor compliance to atropine use, patients with any systemic diseases, with astigmatism >2D, with uniocular progressive myopia, patients using other forms of anti-myopia therapies earlier (orthokeratology/any dose of atropine/pirenzepine), with any retinal pathology or other ocular morbidities and the patients who were lost to follow up at subsequent follow-up visits. A total of 87 children were randomly assigned to two groups (Gr A: 44 children and Gr B: 43 children), and the participants were unaware of group assignment (single blind). 88 eyes of 44 children in group A received once-daily dosing of 0.01% atropine at bedtime, while 86 eyes of 43 children in group B received once-daily dosing of a placebo eyedrop(preservative-free carboxymethyl cellulose 0.5%) at bedtime. Atropine 0.01% eye drops are commercially available in the market, which were used in the study. The study participants underwent a comprehensive ocular examination before carrying out the study, including best-corrected visual acuity (BCVA), cycloplegic refraction using 1% atropine, axial length (AXL) measurements using the intraocular lens (IOL) master 500, slit-lamp examination, dilated fundoscopy, and optical coherence tomography (OCT+HRA Spectralis -Heidelberg). The compliance with daily medication use was monitored with the help of a daily diary maintained by the parents. The parents ensured that they had kept the proper drop count while instilling the drop. All the participants were examined at the same time of day to minimize the influence of circadian fluctuations on choroidal thickness. Spherical equivalence was calculated as the sum of spherical power plus half of the cylindrical power. Sub-foveal choroidal thickness and choroidal thickness at 1500 and 3000 microns from the center of the fovea were documented in all four quadrants (superior, inferior, nasal, and temporal) at baseline,1 month,3 months, and 6 months. All the participants were followed up for a minimum duration of 6 months. All the measurements were done by a single observer to minimize inter-observer variability.

Statistical analysis

Summarizing categorical data into counts and percentages was the first step in statistical analysis. Using the Shapiro-Wilk test, it was possible to ascertain if continuous variables were normally distributed. Mean values with standard deviations (mean ± SD) were used to display variables that had a normal distribution. Results that did not fit the normal distribution were displayed as medians and their interquartile ranges (IQR; 25th to 75th percentiles). For properly distributed continuous data, independent sample t-tests were used to compare groups; for data that did not match normality, the Mann-Whitney U test was utilized. The Wilcoxon signed-rank test or the paired t-test was used for comparisons within the same group (paired data) according to the variable distribution. The Fisher's exact test was used to compare categorical variables, especially when any predicted cell count was fewer than five. Microsoft Excel was used to enter data, and IBM SPSS Statistics, version 25.0 (IBM Corp., Armonk, NY, USA), was used for all statistical analyses. Statistical significance was defined as a p-value of less than 0.05.

## Results

The mean ages were comparable in cases and control groups (10.91+/-2.81 in cases and 10.93+/- 2.82 in controls). There was a slight female preponderance in both the case and control groups, but this was not significant. A significant reduction in mean spherical equivalence (0.5D and 0.88 D) and AXL (0.21 mm and 0.32 mm) were noted in the cases group as opposed to significant increase in the mean spherical equivalence (0.75 D and 0.87 D) and AXL (0.52 mm and 0.74 mm) in the control group at 3 and 6 months of follow-up. The changes in spherical equivalence over time are detailed in Table 1.

**Table 1:** Comparison of changes in spherical equivalence in cases versus controls at presentation and subsequent follow-up visits Diopter(D), Values presented as median (interquartile range). ^§^P-value calculated using the Mann–Whitney U test. *Indicates statistically significant difference at p < 0.05.

Spherical Equivalent (D)				
Time point	Cases (n=88)	Controls (n=86)	Total	P-value
At presentation	-6.5 (-9.125 to -3.212)	-6.75 (-9.5 to -3.25)	-6.75 (-9.625 to -3.25)	0.348^§^
At 1 month	-6.5 (-9.125 to -3.212)	-6.75 (-9.5 to -3.25)	-6.5 (-9.625 to -3.25)	0.348^§^
At 3 months	-6 (-8.625 to -3)	-7.5 (-10.5 to -3.312)	-6.75 (-10 to -3.25)	0.026*^§^
At 6 months	-5.62 (-8 to -3)	-7.62 (-11.0 to -3.5)	-6.88 (-9.938 to -3.152)	0.002*^§^

The changes in AXL are presented in Table 2. The table shows AXL changes in cases and controls over time. While cases remained stable or slightly decreased, controls showed progressive increases. Significant differences at 3 and 6 months suggest intervention effects (p<0.05).

**Table 2:** Axial length changes over time in cases vs controls Values expressed as mean ± standard deviation (SD) ^‡^P-value calculated using an independent t-test. *Indicates statistically significant difference at p < 0.05.

Time Point	Cases (n=88)	Controls (n=86)	Total	P-value
At presentation	25.83 ± 1.76	25.87 ± 1.78	25.96 ± 1.79	0.333^‡^
At 1 month	25.83 ± 1.76	25.87 ± 1.78	25.96 ± 1.79	0.333^‡^
At 3 months	25.62 ± 1.75	26.39 ± 1.89	25.91 ± 1.83	0.036*^‡^
At 6 months	25.51 ± 1.75	26.61 ± 1.89	25.85 ± 1.84	0.013*^‡^

Low-dose atropine (0.01%) significantly increased the sub-foveal choroidal thickness (3.05 microns and 5.02 microns) as well as choroidal thickness in all four quadrants in cases at 3 and 6 months of follow-up respectively whereas sub-foveal choroidal thickness (2.51 and 6.09 microns) and choroidal thickness in all quadrants showed significant thinning in controls at 3 and 6 months follow-ups (Table 3).

**Table 3:** Comparison of sub-foveal choroidal thickness between cases and controls at presentation and subsequent follow-ups. Values expressed as mean ± standard deviation (SD) ‡P-value calculated using an independent t-test. *Indicates statistically significant difference at p < 0.05.

Time Point	Cases (n=88)	Controls (n=86)	Total	P-value
At presentation	295.36 ± 37.63	295.27 ± 36.18	292.84 ± 36.9	0.364^‡^
At 1 month	296.25 ± 38.56	295.27 ± 36.18	294.81 ± 37.57	0.115^‡^
At 3 months	298.35 ± 37.53	292.76 ± 35.56	292.58 ± 37.61	0.018*^‡^
At 6 months	300.38 ± 38.13	288.16 ± 34.04	290.06 ± 37.52	0.0002*^‡^

**Figure 1:**
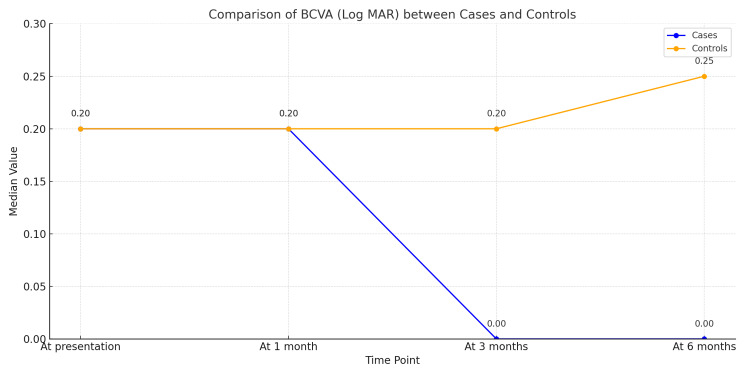
Comparison of the trend of BCVA (Log MAR) between cases and controls at presentation and follow-ups BCVA: best-corrected visual acuity

A significant improvement in BCVA was noted in cases (from 0.2 to 0 logMAR) as compared to a significant decrease in BCVA in controls (from 0 to 0.25 logMAR) at final follow-up (Figure 1).

None of the study participants had any adverse effects following atropine use.

## Discussion

Atropine primarily exerts its effect on pre-junctional M2 and M4 muscarinic receptors located at the cholinergic-nitrergic nerve endings within the choroid. These receptors are crucial for regulating the cerebral nitric oxide signaling pathway, which in turn controls the dilation of blood vessels in the eyes. This mechanism contributes to changes in choroidal thickness, which is believed to influence ocular growth. Notably, higher concentrations of atropine have been linked to causing a rebound effect where myopia progression accelerates within a year after cessation of treatment. In contrast, lower concentrations are generally less likely to trigger this response [19]. The ATOM 2 study found that using a 0.01% concentration of atropine resulted in minimal rebound and provided sustained control over myopia progression over five years [20]. Supporting this, Gong et al. observed a gradual increase in sub-foveal choroidal thickness beginning at the third month of treatment with 0.01% atropine, continuing through the 12-month follow-up [21]. Further evidence was provided by the LAMP study, a rigorously designed double-blind, placebo-controlled randomized clinical trial. This trial assessed the effects of daily atropine at concentrations of 0.05%, 0.025%, and 0.01%. After one year, the mean changes in spherical equivalence (SE) were −0.27 (0.61) D, −0.46 (0.45) D, −0.59 (0.61) D, and −0.81 (0.53) D, respectively. Corresponding AXL changes were 0.20 (0.25) mm, 0.29 (0.20) mm, 0.36 (0.29) mm, and 0.41 (0.22) mm. These outcomes revealed a clear dose-response relationship, with the 0.05% atropine dosage proving to be the most effective in slowing both myopia progression and axial elongation. Compared to placebo, 0.01% atropine reduced axial elongation by 12% and slowed myopia progression by 27% [22]. Side effects were generally mild, and low-dose atropine was well tolerated [22]. Similarly, a study by Ye et al. reported a measurable increase in choroidal thickness at three months following administration of 0.01% atropine, followed by a reduction by the sixth month [23]. Our study aligns with these previous findings, demonstrating that 0.01% atropine is effective in slowing axial elongation and myopia progression, while transiently increasing choroidal thickness at three and six months. These results support the hypothesis that low-dose atropine can influence choroidal structure and help modulate ocular growth. Importantly, no drug-related adverse effects were observed among our study participants.

Study limitations

This was a single-centered study and thus may not completely represent the entire population. The sample size was small, but modest enough for intergroup comparison. As the minimum duration of the follow-up was six months, the study does not assess the effect of low-dose atropine on long-term myopia progression. Lack of double blinding might have introduced some amount of observer bias. We did not account for factors that influence the evolution and progression of myopia, such as lifestyle, outdoor activities, and near-work involvement. We also did not take into account the rebound effect of the drug after cessation of therapy.

## Conclusions

Low-dose atropine (0.01%) was found to be effective in retarding myopia progression in children aged 5-16 years by increasing the choroidal thickness, decreasing the axial elongation of eyeball and SE, as well as improving the BCVA with no significant adverse events. There is a need for further studies with a larger sample size, longer duration of follow-up, and correlation of changes in choroidal thickness with central corneal thickness, pupil size, and IOP. There is also a need for studying the rebound in myopia progression after cessation of the drug and its correlation with different concentrations of atropine.
